# Cost-effectiveness analysis of aspirin for primary prevention of cardiovascular events among patients with type 2 diabetes in China

**DOI:** 10.1371/journal.pone.0224580

**Published:** 2019-12-02

**Authors:** Minghuan Jiang, Pengchao Li, Joyce Hoi-sze You, Xinglong Zheng, Jizhao Deng, Mingyue Zhao, Liuxin Feng, Yu Fang

**Affiliations:** 1 Department of Pharmacy Administration and Clinical Pharmacy, School of Pharmacy, Xi’an Jiaotong University, Xi’an, China; 2 Center for Drug Safety and Policy Research, Xi’an Jiaotong University, Xi’an, China; 3 School of Pharmacy, Faculty of Medicine, The Chinese University of Hong Kong, Shatin, N.T, Hong Kong, China SAR; 4 Department of Cardiovascular Surgery, The First Affiliated Hospital of Xi’an Jiaotong University, Xi’an, China; 5 Department of Cardiovascular Medicine, Shaanxi Provincial People’s Hospital, Xi’an, China; 6 Department of Pharmacy, The Second Affiliated Hospital of Xi’an Jiaotong University, Xi’an, China; Icahn School of Medicine at Mount Sinai, UNITED STATES

## Abstract

The use of aspirin for primary prevention of cardiovascular disease (CVD) in patients with diabetes mellitus (DM) is associated with lower rates of cardiovascular events but increased risks of bleeding complications. We aimed to examine the cost-effectiveness of aspirin therapy for primary prevention of CVD in Chinese DM patients. A life-long Markov model was developed to compare aspirin therapy (100mg daily) versus no use of aspirin in DM patients with no history of CVD. Model validation was conducted by comparing the simulated event rates with data reported in a clinical trial. Direct medical costs and quality-adjusted life-years gained (QALYs) were the primary outcomes from the perspective of healthcare system in China. Sensitivity analyses were performed to examine the uncertainty of model inputs. Base-case analysis showed aspirin therapy was more costly (USD1,086 versus USD819) with higher QALYs gained (11.94 versus 11.86 QALYs) compared to no use of aspirin. The base-case results were sensitive to the odds ratio of all-cause death in aspirin therapy versus no use of aspirin. Probabilistic sensitivity analysis found that aspirin therapy gained an additional 0.066 QALYs (95% CI: -0.167 QALYs-0.286 QALYs) at higher cost by USD352 (95% CI: USD130-644)). Using 30,000 USD/QALY as willingness-to-pay threshold, aspirin therapy and no use of aspirin were the preferred option in 68.71% and 31.29% of 10,000 Monte Carlo simulations, respectively. In conclusion, aspirin therapy appears to be cost-effective compared with no use of aspirin in primary prevention of CVD in Chinese DM patients.

## Introduction

The prevalence of diabetes mellitus (DM) has increased by 17-fold (0.67% to 11.6%) from 1980 to 2010 in China [1]. DM is associated with a high-risk of cardiovascular disease (CVD), and CVD accounts for almost half of mortality in Chinese DM patients [1, 2]. The standards of care for diabetes in China recommend that DM patients aged over 50 years with cardiovascular risk factors (such as dyslipidemia, smoking, or proteinuria) to receive aspirin therapy for primary prevention of CVD [2].

Although the effectiveness of aspirin for secondary prevention of CVD has been well established worldwide, the role of aspirin in primary prevention is still controversial [3]. Findings from previous meta-analysis and large randomized clinical trials reported that there was no significant reduction of cardiovascular events in healthy elderly or patients at low-to-moderate cardiovascular risk who received aspirin for primary prevention of CVD [4–6]. However, the clinical benefits and hazards of high-risk patients taking aspirin for primary prevention were uncertain [7].

The ASCEND (A Study of Cardiovascular Events iN Diabetes) Trial [8] was the largest randomized trial (N = 15,480) examining the clinical outcomes of aspirin use for primary prevention of CVD in DM patients. DM patients who had no history of CVD were randomized to receive low-dose aspirin (100mg daily) or placebo with a mean follow-up of 7.4 years. Results showed that the rates of serious vascular events (myocardial infarction (MI), stroke or transient ischemic attack (TIA), or vascular death) were significantly lower in the aspirin group (8.5%) than the placebo group (9.6%), yet higher major bleeding events occurred in the aspirin group (4.1% versus 3.2%). Similar findings were reported by a meta-analysis of ten randomized trials concluding that the net benefits of taking aspirin in DM patients were largely counterbalanced by the increased risks of gastrointestinal (GI) bleedings [9]. Recent clinical practice guideline of American College of Cardiology and American Heart Association on primary prevention of CVD recommended that low-dose aspirin (75-100mg daily) might be considered for primary prevention in adults aged 40–70 years who were at high-risk of CVD [10]. DM patients were at high 10-year CVD risk, therefore, a quantitative assessment of the clinical benefits and risks of aspirin use for primary prevention in DM patients was highly warranted.

Several studies have reported the findings of pharmacoeconomic evaluations on aspirin use for primary prevention in patients with various risks of CVD [11]. Low-dose aspirin use was consistently found to be cost-effective compared to no use of aspirin. However, the previous cost-effectiveness studies were mainly applicable to general subjects of Western populations and rare studies specifically focused on DM patients or the Chinese population. In the present study, we aimed to evaluate the clinical and economic outcomes of aspirin use for primary prevention of CVD in Chinese DM patients.

## Methods

### Decision-analytic model

**Fig 1:**
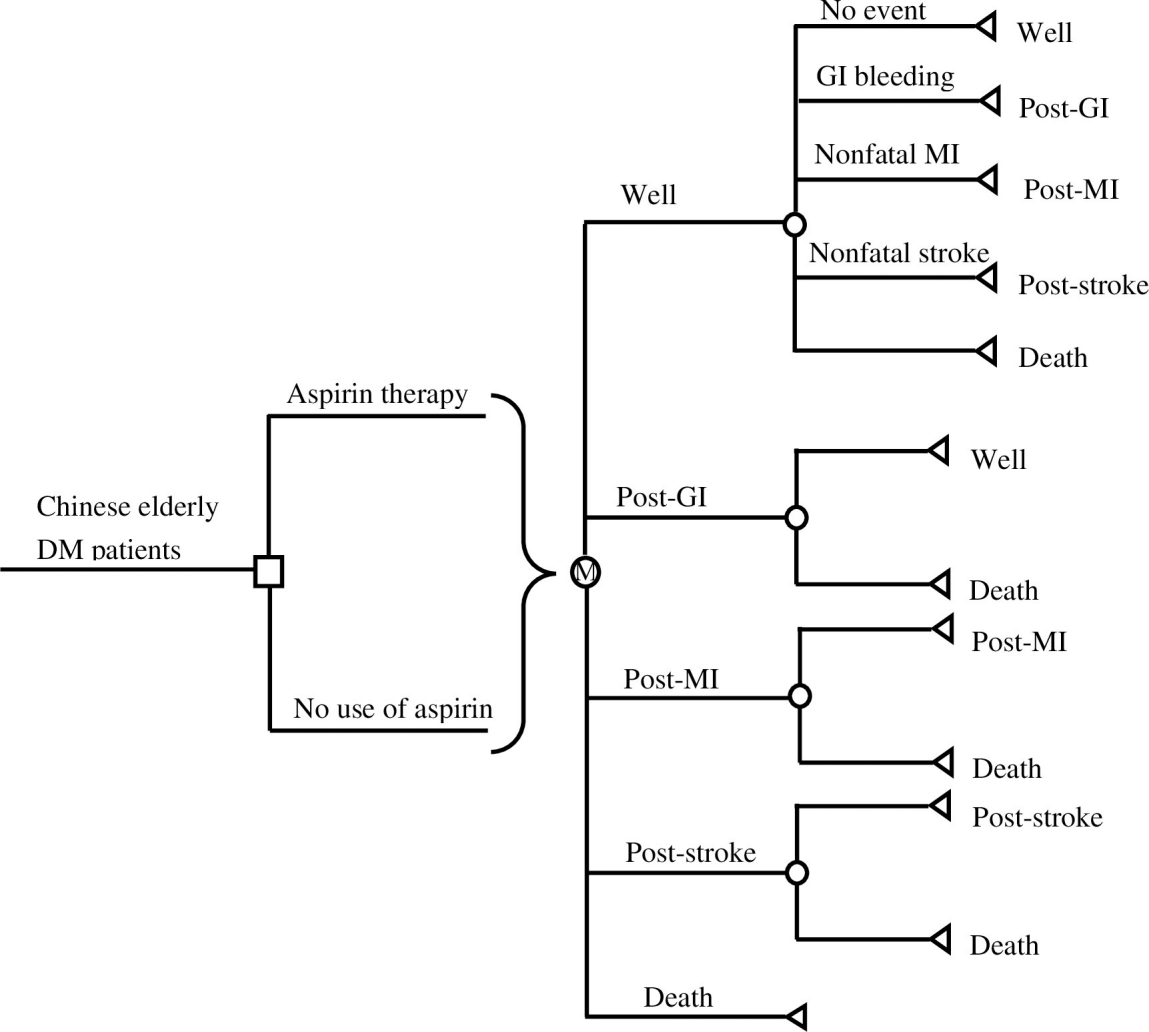
Simplified decision-analytic model of aspirin therapy versus no use of aspirin for primary prevention of CVD in DM patients. CVD: cardiovascular disease; DM: diabetes mellitus; GI: gastrointestinal; MI: myocardial infarction.

We designed a life-long Markov model (**Fig 1**) to simulate aspirin therapy (100mg daily) and no use of aspirin for primary prevention of CVD in a hypothetical cohort of 60-year-old DM patients. The population age was similar to the mean age (63 years) of patients in the ASCEND Trial [8]. Patients included in the model were those with no known occlusive arterial disease, and patients who had a history of bleeding or recent surgical operation were excluded. The primary characteristics of patients simulated in the present study were consistent with those who participated in the ASCEND Trial [8]. Direct medical costs and quality-adjusted life-years gained (QALYs) were the primary outcomes simulated for each option in the model from the perspective of healthcare system in China.

The present model simulated transitions of five health states for a maximum of 40 years with yearly cycle. The health states included well, post-GI bleeding, post-MI, post-stroke, and all-cause death. All patients would initially start at the well state, and they might experience no event, GI bleeding, nonfatal MI, nonfatal (ischemic or hemorrhagic) stroke, or die of cardiovascular or non-cardiovascular causes during the first year. For individuals with GI bleeding, they would die or transit to the post-GI bleeding state. Patients in the post-GI bleeding state were assumed to discontinue use of aspirin, and survivors would transit to the well state within one year. Patients who experienced nonfatal MI or nonfatal stroke would transit to the post-MI or post-stroke state in the following year. Patients in the post-MI or post-stroke state would survive at this health state until they died.

### Clinical inputs

Clinical inputs are shown in **Table 1**. We used the keywords ‘diabetes mellitus’, ‘aspirin’, ‘primary prevention’, ‘cardiovascular events’, ‘myocardial infarction’, ‘stroke’, ‘bleeding’, and ‘QALY’ to search published literature on Medline and Embase within the period of 2000–2019. The selection criteria of relevant studies were: 1) published in English; 2) primary or sub-group population included were DM patients with no history of CVD; 3) aspirin was used for primary prevention of cardiovascular events; 4) clinical event rates and corresponding odds ratios or relative risks were reported. Meta-analyses and randomized clinical trials were the preferred sources of clinical inputs.

**Table 1:** Model inputs.

	Base-case value	Range	Distribution type	Reference
**Clinical Inputs**				
Annual event rate in DM patients with no use of aspirin				
Nonfatal MI	0.0031	0.0025–0.0037	β	[8, 12]
Nonfatal stroke	0.0042	0.0038–0.005	β	[8, 12]
GI bleeding	0.0018	0.0014–0.0022	β	[8, 12]
All-cause death/Age groups (years)				[14]
60 to 64	0.0133	-	-	
65 to 69	0.0234	-	-	
70 to 75	0.0356	-	-	
75 to 79	0.0529	-	-	
80 to 84	0.1159	-	-	
≥85	0.2263	-	-	
Odds ratio of aspirin therapy versus no use of aspirin				
Nonfatal MI	0.98	0.93–1.04	Lognormal	[8]
Nonfatal stroke	0.88	0.73–1.06	Lognormal	[8]
GI bleeding	1.57	1.37–1.80	Lognormal	[15]
Death	0.94	0.85–1.04	Lognormal	[15]
Discontinuation rate of aspirin therapy	0.3	0.24–0.36	β	[8]
Adjusting factor on clinical event rates	1	0.5–2	-	Assumption
Probability of health state transition				
Post-MI to death (year 1)	0.188	0.15–0.24	β	[16]
Post-Stroke to death (year 1)	0.144	0.115–0.173	β	[17]
Post-MI/Post-stroke to death (subsequent years)	0.07	0.05–0.09	β	[16, 18]
Post-GI bleeding to death	0.03	0.024–0.036	β	[19, 20]
Length of hospitalization days for major bleeding	15	13–18	Uniform	[23]
**Utility Inputs**				
Elderly DM patients	0.876	0.701–1	Uniform	[21]
Disutility of GI bleeding	0.25	0.188–0.313	Uniform	[22]
Disutility of post-MI	0.139	0.111–0.167	Uniform	[21]
Disutility of post-stroke	0.215	0.172–0.258	Uniform	[21]
Disutility of taking aspirin	0.001	0.0008–0.0012	Uniform	[20]
Cost Inputs* **(USD)**				
Monthly cost of aspirin 100mg daily	2.4	1.9–2.9	Lognormal	Local price
GI bleeding	3,443	2,754–4,132	Lognormal	[23]
Nonfatal MI	4,346	3,477–5,216	Lognormal	[14]
Nonfatal stroke	1,508	1,446–2,860	Lognormal	[14]
Yearly cost of post-MI	500	317–683	Lognormal	[24]
Yearly cost of post-stroke	556	490–909	Lognormal	[24]

DM: diabetes mellitus; MI: myocardial infarction; GI: gastrointestinal; 1USD = 6.61 Chinese Yuan.

*The base-year for cost inputs is the year 2019.

The annual event rates of nonfatal MI, nonfatal stroke, and GI bleeding in DM patients with no use of aspirin were estimated from the pooled data reported in the ASCEND Trial [8] and the JPAD (Japanese Primary Prevention of Atherosclerosis with Aspirin for Diabetes) Trial [12]. The JPAD Trial examined the clinical outcomes of aspirin for primary prevention of atherosclerotic events in 2,539 Japanese DM patients with a follow-up of 4.37 years. The annual event rate in each trial was estimated using the following formula: *r = -[ln(1-p)]/t*, where p was the accumulated event rate reported at time t in the trial [13]. Since there was no substantial difference of participants’ key characteristics in the above trials, the pooled data was further estimated by calculating the weighted average event rate from different trials according to the number differences of participants in each trial. The age-specific all-cause mortality rates in DM patients were estimated from the 2017 life table reported in the China Health Statistics Yearbook [14]. The event rates in aspirin therapy arm were further calculated by the data in patients with no use of aspirin and corresponding event odds ratios of aspirin therapy versus no use of aspirin. The odds ratios for nonfatal MI and nonfatal stroke were retrieved from the ASCEND Trial [8]. The odds ratios for GI bleeding and all-cause death were derived from the DM subgroup analysis in a meta-analysis of 11 clinical trials examining the efficacy and safety of aspirin use for primary prevention of CVD [15]. The discontinuation rate of taking aspirin (30%) was estimated from the finding reported in the ASCEND Trial [8].

The probabilities of post-MI state to death in the first year and subsequent years were adapted from the model designed by Main et al. using surviving data from the Nottingham Heart Attack Register [16]. The probability of post-stroke to death in the first year was estimated from a national registry study recruiting 12,415 patients with ischemic stroke in China [17]. The probability of post-stroke to death in subsequent years was assumed to be similar to that of post-MI to death [18]. The mortality rate of GI bleeding was estimated at 3%, which was derived from previous cost-effectiveness analyses of aspirin use for primary prevention of CVD [19, 20].

### Utility and costs inputs

The utility score of elderly DM patients and disutility scores of post-MI and post-stroke were derived from a questionnaire survey with the EQ-5D-5L instrument on an assessment of the health-related quality of life in 289 Chinese patients with type 2 diabetes [21]. The disutility of GI bleeding was derived from a risk-benefit analysis on an assessment of alternative antiplatelet strategies in patients with the percutaneous coronary intervention [22]. The disutility of taking aspirin was derived from a cost-effective analysis of aspirin treatment for primary prevention in elderly patients with CVD [20]. The utility values of post-MI and post-stroke state in DM patients were further estimated by the sum of utility of elderly DM patients and their corresponding disutility. The QALYs gained in each health state were calculated by using the utility of the health state and life-years spent in this state. The QALYs loss from GI bleeding was estimated by the disutility of bleeding multiplied by the length of hospitalization of GI bleeding. The QALYs loss from aspirin taking was calculated by the disutility of aspirin taking multiplying the years for aspirin medication. The total QALYs gained in each option of the model were the summation of QALYs of all health states experienced by the patients.

Direct medical costs were included in the present study for cost analysis (1USD = 6.61 Chinese Yuan). The monthly cost of generic aspirin (USD2.4; range: USD1.9–2.9) was retrieved from public hospitals in China. The treatment cost and length of stay (15 days; range: 13–18 days) of GI bleeding were derived from a retrospective study (N = 312) on assessing hospitalization costs of bleeding events by using medical records data of seven tertiary hospitals in China [23]. The costs of nonfatal MI and nonfatal stroke were derived from the China Health Statistic Yearbook [14], which were the one-time direct medical costs for management of MI and stroke events. The yearly costs of post-MI and post-stroke were derived from previous cost-effectiveness studies of statin treatment in Chinese DM patients [24], which were used to calculate the annual treatment costs of survivors from MI and stroke events. We assumed that the cost of aspirin medication was included in the yearly management cost of post-MI or post-stroke, whilst no aspirin medication cost occurred for patients in the post-GI bleeding state within the year. All costs from published literature were adjusted to 2019 data by using the inflation rate of the annual consumer price index of medical care from the National Bureau of Statistics of China [25]. All costs and QALYs simulated in the present study were discounted to the year 2019 with an annual rate of 3%.

### Cost-effectiveness analysis and sensitivity analysis

TreeAge Pro 2018 (TreeAge Software Inc., Williamstown, MA) and Microsoft Excel 2013 (Microsoft Corporation, Redmond, WA, USA) were utilized to perform cost-effectiveness and sensitivity analyses. The life-long costs and QALYs gained in each strategy were reported. The incremental cost-effectiveness ratio (ICER) was calculated when one strategy cost more with higher QALYs gained by using the equation: *Δcost/ΔQALYs*. As recommended by the World Health Organization [26], the strategy was considered to be cost-effective when the ICER was less than 3-fold gross domestic product (GDP) per capita. The GDP per capita in China was obtained from the National Statistics Bureau for the year 2017 [25], which was adjusted to the year 2019 (USD9,860 GDP per capita) using the projected GDP growth rate from 2017 to 2019 reported by the International Monetary Fund [27]. Therefore, the willingness-to-pay (WTP) threshold applied in the present study was 30,000 USD/QALY (3-fold GDP per capita). The most effective strategy with ICER less than the WTP threshold was considered to be a cost-effective or preferred option.

Sensitivity analyses were conducted to examine the robustness of model results. One-way sensitivity analysis over variable ranges (95% confidence interval (CI) or ±20% of base-case values) was performed to examine the impact of each variable on the base-case results. To evaluate the uncertainty of all variables simultaneously, probabilistic sensitivity analysis was performed with 10,000 Monte Carlo simulations by drawing each model input from specific probability distribution specified in **Table 1**.

## Results

### Model validation and base-case analysis

To examine the predictive validity of the model, we compared the simulated 7-year event rates in each strategy with findings reported in the ASCEND Trial (mean follow-up of 7.4 years). The simulated event rates of bleeding, nonfatal MI, nonfatal stroke, and all-cause death in both arms of the model were all within ±10% relative difference comparing to the data from the ASCEND Trial (**Table 2**).

**Table 2:** Model validation.

	Aspirin therapy			No use of aspirin		
	Trial*	Model	Difference	Trial*	Model	Difference
GI bleeding	0.018	0.0186	3.3%	0.013	0.012	-7.7%
Nonfatal MI	0.025	0.0226	-9.6%	0.025	0.023	-8.0%
Nonfatal stroke	0.026	0.0244	-6.15%	0.03	0.0298	-0.67%
All-cause death	0.097	0.1065	9.8%	0.102	0.1073	5.2%

* the ASCEND Trial; GI: gastrointestinal; MI: myocardial infarction.

The base-case results were shown in **Table 3**. Aspirin therapy was more costly (USD1,086 versus USD819) with higher QALYs gained (11.94 versus 11.86 QALYs) compared to no use of aspirin. Aspirin therapy was a cost-effective option with ICER of 3,338 USD/QALY below the WTP threshold (30,000 USD/QALY).

**Table 3:** Base-case results.

	Cost (USD)	Incremental Cost	QALYs	Incremental QALYs	ICER (USD/QALY)
No use of aspirin	819	-	11.86	-	-
Aspirin therapy	1,086	267	11.94	0.08	3,338

QALY: quality-adjusted life-years; ICER: incremental cost-effectiveness ratio.

### Sensitivity analysis

One-way sensitivity analysis on all model variables found the base-case results to be sensitive to the odds ratio of all-cause death (0.94; range: 0.85–1.04) of aspirin therapy versus no use of aspirin. No use of aspirin would become cost-effective when the odds ratio of all-cause death exceeded 1.001.When the odds ratio of all-cause death was lower than 1.001, aspirin therapy gained more QALYs at higher cost with ICERs below the WTP threshold (30,000 USD/QALY), therefore, aspirin therapy was the cost-effective strategy. When the odds ratio varied between 1.001 and 1.003, the ICERs of aspirin therapy versus no use of aspirin exceeded the WTP threshold, and no use of aspirin became a cost-effective option. When the odds ratio was over 1.003, the aspirin group was dominated by no use of aspirin because aspirin therapy became less effective (lower QALYs gained) and more costly.

In base-case scenario, we examined a disutility of 0.001 in DM patients taking aspirin on the impact of their quality of life. If no disutility of taking aspirin were considered, the additional QALYs gained by aspirin therapy would be higher with lower ICER (2,883 USD/QALY), and aspirin therapy would be more cost-effective compared to no use of aspirin. The present study examined the cost-effectiveness of aspirin therapy in a cohort of 60-year-old DM patients. The clinical event rates of DM patients with or without aspirin therapy increased with age. To further examine the impact of age effects on base-case results, we extended the variable ranges by adding one adjusting factor (1; range: 0.5–2) to all clinical event rates on nonfatal MI, nonfatal stroke, GI bleeding, and all-cause death simultaneously. Extended one-way sensitivity analysis found the base-case results were robust that aspirin therapy remained cost-effective throughout the variation of the adjusting factor. The ICERs of aspirin therapy varied within 2,643–4,592 USD/QALY below the WTP threshold, and the ICERs decreased with the increase of clinical event rates. The findings indicated that aspirin therapy would be more cost-effective in DM patients with older age.

**Fig 2:**
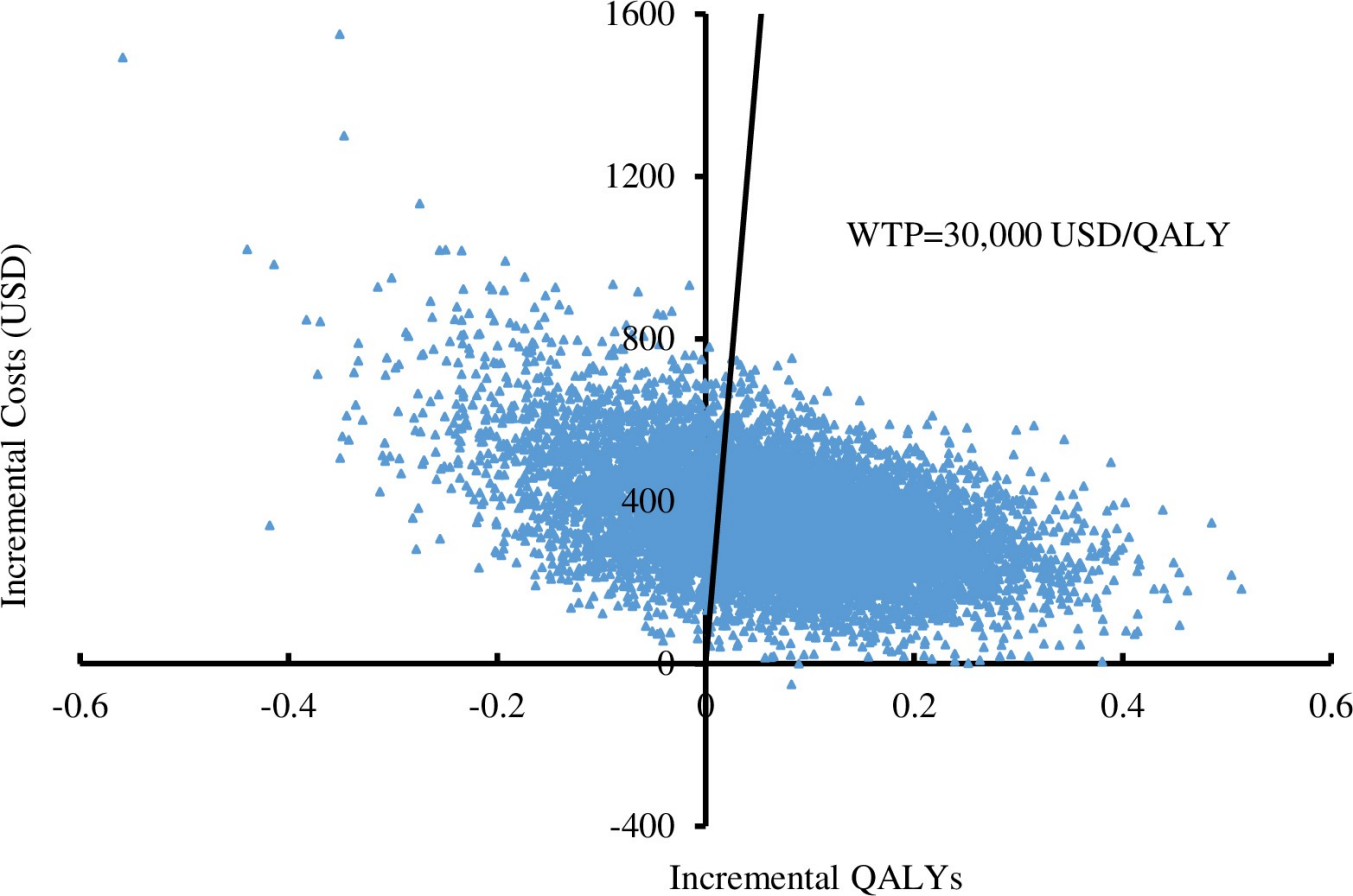
Scatter plots of incremental cost against incremental QALYs of aspirin therapy versus no use of aspirin. QALY: quality-adjusted life-years; WTP: willingness-to-pay.

Probabilistic sensitivity analysis was performed by 10,000 Monte Carlo simulations. Compared to no use of aspirin, aspirin therapy gained more QALYs (11.61 versus 11.54 QALYs) at higher cost (USD1,142 versus USD791). Aspirin therapy was more costly by USD352 (95% CI: USD130-644) with an additional gain of 0.066 QALYs (95% CI: -0.167 QALYs-0.286 QALYs). The 95% CIs of cost and QALYs differences between two strategies were calculated by using percentile method based on ranking differences in 10,000 simulations. Of 10,000 simulations (**Fig 2**), aspirin therapy gained higher QALYs at lower cost in 0.01% of the time. Aspirin therapy was more costly and gained higher QALYs with ICERs below and above the WTP threshold of 30,000 USD/QALY in 68.70% and 3.84% of the time, respectively. The remaining 27.45% simulations showed aspirin therapy to be less effective (lower QALYs gained) compared to no use of aspirin. Given the uncertainty of all model inputs, the expected value of perfect information was USD744 by using 30,000 USD/QALY as the WTP threshold, and further research might be needed to reduce the uncertainty.

**Fig 3:**
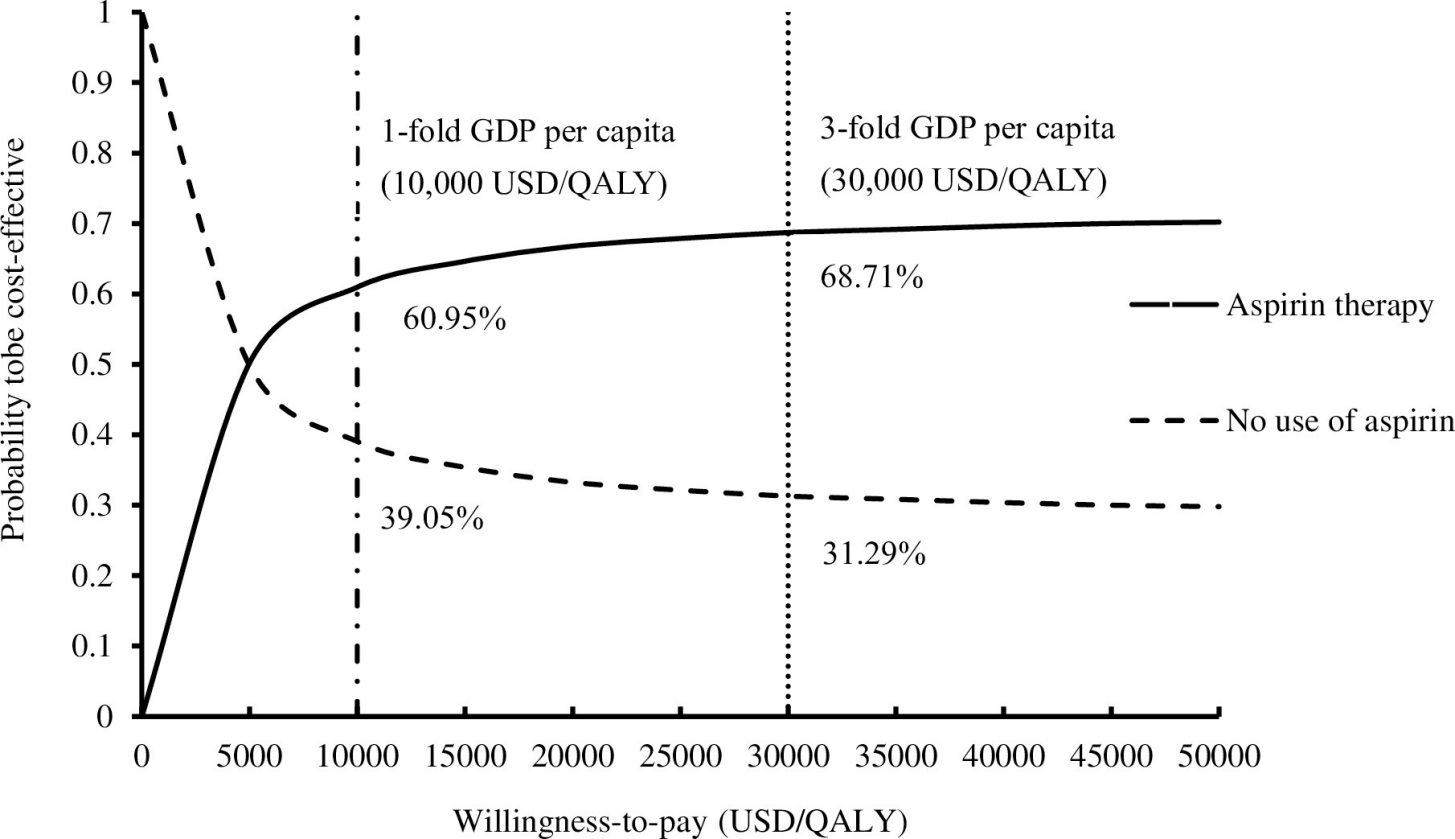
Variation in probability of each therapy to be cost-effective against WTP per QALY. QALY: quality-adjusted life-years; GDP: gross domestic product; WTP: willingness-to-pay.

The probabilities of each strategy to be cost-effective were examined in the acceptability curve over the range of 0–50,000 USD/QALY as the WTP threshold (**Fig 3**). Using 3-fold GDP per capita as the WTP threshold (30,000 USD/QALY), the probability of aspirin therapy and no use of therapy to be cost-effective were 68.71% and 31.29%, respectively. When 1-fold GDP per capita was the WTP threshold (10,000 USD/QALY), the probability of aspirin therapy and no use of aspirin to be the preferred option were 60.95% and 39.05%, respectively.

## Discussion

The present study was the first cost-effectiveness analysis to evaluate aspirin use for primary prevention in Chinese DM patients. Base-case analysis found that aspirin therapy was a cost-effective option with ICER lower than the WTP threshold when compared to no use of aspirin. Probabilistic sensitivity analysis further supported aspirin therapy to be preferred in 68.71% of 10,000 Monte Carlo simulations when using 3-fold GDP per capita as the WTP threshold (30,000 USD/QALY).

The robustness of base-case results was sensitive to the odds ratio of all-cause death with aspirin therapy versus no use of aspirin. When the odds ratio of all-cause death increased, the QALYs gained in aspirin therapy would decrease dramatically due to a higher mortality rate. The benefits of aspirin therapy versus no use of aspirin would be narrowed and the ICER of aspirin therapy would be higher and exceeded the WTP threshold when the odds ratio of all-cause death was above 1.001. In the present study, the odds ratio of all-cause death (0.98; 95% CI: 0.93–1.04) and GI bleeding (1.57; 95% CI: 1.37–1.80) were derived from the subgroup analysis for diabetes (N = 139,229) in a recent meta-analysis on nine clinical trials [15]. If using data from trials with DM patients only (the ASCEND Trial and the JPAD Trial, N = 18,019) [15], the two odds ratios (0.94 and 1.27 for all-cause death and bleeding, respectively) were also within the variable range that aspirin therapy could be cost-effective.

Although only a few studies evaluated the cost-effectiveness of aspirin use for primary prevention in DM patients, previous studies consistently demonstrated that aspirin use was a cost-effective option in a variety of scenarios [11, 19, 20, 28–31]. One study from the US showed a statewide campaign in Minnesota to promote regular aspirin use in primary prevention would avert 9,874 MI events in men and 1,223 stroke events in women [19]. Although the campaign was associated with 7,222 more major GI bleedings, the promotion of aspirin use was found to be cost-saving at higher QALYs gained than the current standard of practice. Another study examined the cost-effectiveness of aspirin use for primary prevention in subgroups based on age, gender, and various levels of CVD risk, and results revealed that aspirin use is cost-effective for men with a 10-year CVD risk of >10% and women with a risk of >15% [20]. In our study that based on the results from the ASCEND Trial and recent meta-analysis, we added insights on balancing the ischemic and bleeding risks of aspirin use for primary prevention in elderly DM patients with no history of CVD.

Most of the previous clinical trials and meta-analyses failed to prove the net benefits of aspirin use in DM patients, mainly because the trials were underpowered and had small number of participants included with different defined primary outcomes [3]. The recommendations of aspirin use for primary prevention were conflicting in guidelines provided by different countries. The U.S. Preventive Services Taskforce recommended adults aged 50–69 years with a 10-year CVD risk of ≥10% to initiate low-dose aspirin use, whilst there was no recommendation of aspirin use for adults <50 years or ≥70 years due to insufficient evidence on its balance of benefits and harms [32]. The high-risk factors of 10-year CVD events included older age, male sex, ethnicity, abnormal lipid levels, high blood pressure, smoking, and diabetes [32]. The American Diabetes Association and the American Heart Association recommended aspirin for DM patients at high-risk, whilst the European Society of Cardiology did not recommend [33, 34]. The Chinese guidelines for primary prevention of CVD were consistent with the US guidelines [2]. The ASCEND Trial was the largest and expecting trial to shed light on the benefits and hazards of aspirin use for primary prevention in DM patients. Although the 12% risk reduction of serious vascular events at 7.4-year follow-up in aspirin use seemed largely offset by the 29% increase of bleeding complications, the life-long impacts of the clinical outcomes caused by aspirin use were still uncertain. Our study therefore developed a life-long model to quantitatively evaluate the clinical and economic outcomes of aspirin use for primary prevention in DM patients. The findings of our study would inform decision-makers on how to apply aspirin use in a healthcare system cost-effectively. In clinical practice, gastro-protective agents can be used to reduce the rates of bleedings, especially in the upper gastrointestinal tract. The use of gastro-protective agents together with aspirin therapy would potentially increase the cost-effectiveness of aspirin use for primary prevention in DM patients.

There are several limitations to the present study. Firstly, model-based study is generally subject to the uncertainty of model parameters. The annual event rates of DM patients with no use of aspirin were not available specifically for a Chinese population. We assumed the clinical outcomes of aspirin use had high transferability among countries; therefore, the data were mainly retrieved from large clinical trials in the Western population. We assumed constant annual event rates of nonfatal MI, nonfatal stroke, and GI bleeding in the Markov model, which underestimated the ischemic and bleeding risks in DM patients with increasing age. Rigorous sensitivity analyses were performed to examine the uncertainty of all model inputs. The results of the present study would be updated when the event rates were available from China in the future. Secondly, in the ASCEND Trial, TIA was one of the serious vascular events, and we did not simulate TIA as a separate health state because they were minor and transient, which could be considered as part of survival events before nonfatal stroke. Thirdly, the ASCEND Trial found that there were no benefit on risk reduction of GI tract cancer or any other cancer despite over seven years of aspirin use, which was in contradiction to the previous results of meta-analyses of clinical trials on aspirin treatment [8]. Thus, we did not include cancer as one of the clinical outcomes in the Markov model of the present study. Earlier studies including cancer effects as clinical outcomes consistently found aspirin therapy was cost-effective compared to no use of aspirin for primary prevention [35, 36]. If long-term benefits of aspirin use in reducing cancer risks were confirmed, aspirin therapy for primary prevention of CVD to be cost-effective would be further supported. Lastly, our study was carried out from the perspective of healthcare system. Therefore, the cost-analysis was limited to including direct medical costs only. Diabetic-related complications would require additional outpatient or inpatient care, resulting in loss of productivity and missed working days, especially for younger DM patients. The cost analysis would include both direct and indirect costs if taking societal perspective into account. However, since the cohort simulated in our study was 60-year-old elderly, the indirect costs from productivity loss would be minimal.

In conclusion, aspirin therapy for primary prevention of CVD in Chinese DM patients appears to be cost-effective compared to no use of aspirin. The cost-effectiveness of aspirin therapy was subject to the odds ratio of all-cause death and odds ratio of nonfatal stroke with aspirin therapy versus no use of aspirin.

## Supporting information

S1 Appendix Supporting information for 10,000 Monte Carlo simulations. (XLS)
